# Three New Reports of *Trichoderma* in Algeria: *T. atrobrunneum*, (South) *T. longibrachiatum* (South), and *T. afroharzianum* (Northwest)

**DOI:** 10.3390/microorganisms8101455

**Published:** 2020-09-23

**Authors:** Sadika Haouhach, Noureddine Karkachi, Bouchra Oguiba, Abouamama Sidaoui, Isabel Chamorro, Mebrouk Kihal, Enrique Monte

**Affiliations:** 1 Applied Microbiology Lab, University Oran 1 Ahmed Ben Bella, 31000 Oran, Algeria; noureddinekarkachi@hotmail.com (N.K.); robadz@hotmail.fr (B.O.); kihalm@gmail.com (M.K.); 2 Department of Biotechnology, University of Science and Technology of Oran Mohamed Boudiaf, 31000 Oran, Algeria; 3 Department of Biology, University Center of Tamanrasset, 11000 Tamanrasset, Algeria; sidaouibouamama@gmail.com; 4 Department of Microbiology and Genetics, Spanish-Portuguese Institute for Agricultural Research (CIALE), University of Salamanca, 37185 Salamanca, Spain; isachamorro@usal.es (I.C.); emv@usal.es (E.M.)

**Keywords:** *Trichoderma*, ITS, *tef1*, Algeria, phylogenetic

## Abstract

The genus *Trichoderma* (Hypocreaceae, Ascomycota) consists of globally distributed fungi. In Algeria, few studies have explored the diversity of this genus, and in the majority of works identification is based on phenotypic characters. Here, nine *Trichoderma* strains were collected from Algeria in different locations, namely: seven in the south and two in the northwest. Also, we used 17 reference strains that were taken from the NCBI database for the phylogeny analysis. Our study is based on an integrated approach using micro and macro phenotypic characters and multiple DNA analysis (internal transcribed spacer (ITS): ITS1–4 region; translation elongation factor 1: *tef1* gene). Our study reports, for the first time, three species of *Trichoderma* in Algeria, namely: *T. atrobrunneum* (south), *T. longibrachiatum* (south), and *T. afroharzianum* (northwest). It is noteworthy that *T. atrobrunneum* is a species previously described in European Mediterranean countries, and its presence in the soil of southern Algeria indicates that the diversity of the geographic environments and different climates of Algeria offers the possibility for the survival of diverse *Trichoderma* species. Knowledge on the diversity of these fungi may contribute to their future exploitation in biotechnological applications and to the biological control of plant diseases.

## 1. Introduction 

The ascomycetous genus *Trichoderma* (teleomorph: *Hypocrea*) is one of the most important filamentous fungi frequently found in agricultural habitats because of their ability to colonize the rhizosphere and to progress in different soils from distinct geographic areas [1,2]. Many *Trichoderma* strains can be used as effective biocontrol agents to directly control ascomycetes, basidiomycetes, oomycetes, and nematodes, and some *Trichoderma* metabolites can eben inhibit the growth of certain bacteria [3,4]. In the genome, *Trichoderma* spp. have the potential to produce a huge array of fungal cell wall degrading enzymes (CWDE), such as glucanases, chitinases, and proteases [5]; secondary metabolites with antibiotic activity, such as pyrones, trichothecenes, polyketides, and peptaibols [6,7]; and a high number of genes encoding chaperones and ATP-binding cassette (ABC) transporters that provide them with a great environmental opportunity to repair cell damage, facilitate nutrition, perform detoxification work, and therefore compete in the soil [8]. *Trichoderma* spp. are also good competitors in the rhizosphere as they have the particularity of tolerating high levels of reactive oxygen species (ROS) [9] and can induce ROS production for their own propose, which in turn facilitates the CWDE activity against pathogens [10]. These traits connect the mycoparasitism and the strategies to make it happen, as in the ancestral lifestyle of *Trichoderma* [8]. Comparative genomics studies have suggested that, in a subsequent evolutionary event, the *Trichoderma* lifestyle changed towards root colonization, endophytism, and the beneficial and stable relationships with plants [11].

It is a well-documented fact that *Trichoderma* spp. promote the aboveground biomass growth and produce phytohormones that increase root development [12,13], with root system subsequently becoming deeper and stronger, thus providing more tolerance to drought and abiotic stresses. *Trichoderma* spp. also increase the solubilization and uptake of nutrients, encouraging the hydrophobic adherence and the development of lateral root hairs, with the consequent absorption surface increasing [14]. As a result, *Trichoderma* promote seed germination, increase the plant dry mass content, leaf greenness, and photosynthetic efficiency [15]. These beneficial effects of *Trichoderma* to plants give rise to increased plant protection and crop yields [16]. The *Trichoderma*–plant crosstalk acts on the nodes balancing the costs of plant growth and defense [17], resulting in an induction of systemic signals to promote growth and prime defenses against pathogens and abiotic stresses, whose intensity varies over time [18]. This means that *Trichoderma* strains can be used as a bioprotectant or as plant biostimulant in sustainable agriculture [19].

In any case, it is necessary for a correct species characterization before registration and commercialization. Species identification based on traditional phenotypic methods is often time consuming and laborious, making this type of study difficult because of the unstable and subjective nature of the phenotypic characteristics, which are easily influenced by growing conditions [20]. Identification using molecular techniques combines a variety of parameters in order to properly identify species [21]. This allows for phylogenetic comparisons based on target sequences, thus determining the precise relationships between *Trichoderma* spp. [22].

Given the strong interest of applied aspects of *Trichoderma*, a large number of gene sequence-based studies focused on the taxonomy of this hyperdiverse genus have mushroomed over recent years. Older surveys are based on biocontrol abilities [1,23,24], on cultivated fungi green mold attack [25], or on random collections of *Trichoderma* [26]. More recent studies have concentrated on soil-inhabiting species in geographically limited areas. Thus, a large-scale study of sexual and asexual *Trichoderma* morphs collected from plant and fungal materials has been conducted in Southern Europe, including the European Mediterranean Basin and the African islands of Madeira (Portugal) and the Canary Islands (Spain) [27]. However, in Africa, the diversity of *Trichoderma* species remains relatively unexplored compared with other parts of the world. With the present approach, we want to extend former surveys on *Trichoderma* diversity, in which *T. atroviride* and *T. hamatum* were prevalent in cultivated soils from Tunisia [28]; *T. asperellum*, *T. hamatum,* and *T. virens* were found in soil and compost samples in Morocco [29]; and *T. asperellum*, *T. harzianum,* and *T. ghanense* were isolated from tomato crop soils in the north and northeast of Algeria [30].

The aim of this study is to identify novel Algerian *Trichoderma* species based on the morphological characterization described by Bisset [31,32], as well as the internal transcribed spacer (ITS) and translation elongation factor1 (*tef1*) gene sequence alignment [33]. Therefore, we constructed a phylogenetic tree using the concatenated data set of ITS + *tef1* sequences from *Trichoderma* spp. isolated in the northwest and south of Algeria in order to contribute to the previous studies on *Trichoderma* diversity from North African Mediterranean countries.

## 2. Materials and Methods 

### 2.1. Samples

**Figure 1:**
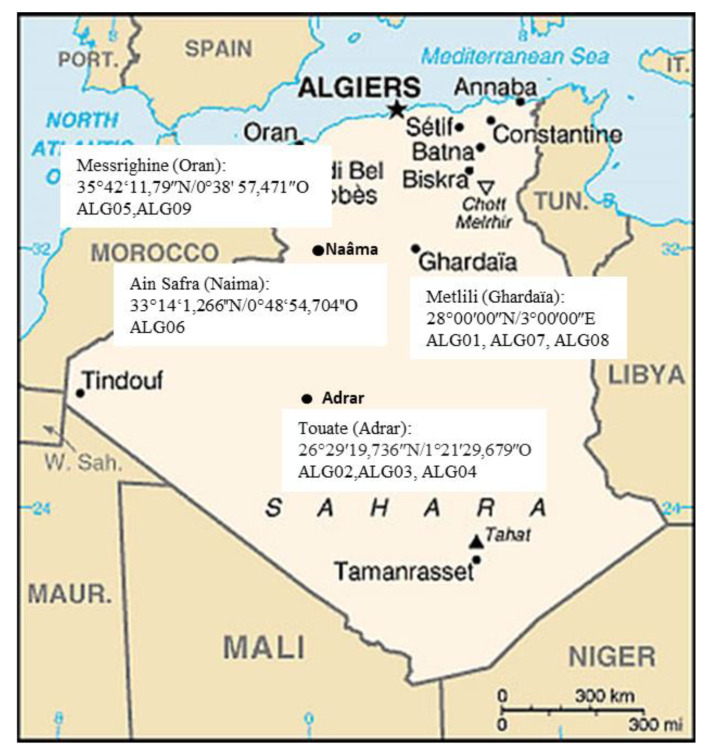
Map of Algeria showing sampling regions of *Trichoderma* isolates during 2015–2016 (Illustration source: https//www.google.dz/maps).

Nine strains of *Trichoderma* were isolated from rhizospheric soils using a dilution plate method. Soil samples were collected from agricultural fields located in different sites of south and northwest in Algeria (Figure 1). Each strain was cultured on a Potato Dextrose Agar (PDA, Difco) medium at 28 °C, and stored at −80 °C in a 20% glycerol solution.

### 2.2. Macroscopic Study

Morphological identification was done based on cultural (colony and growth rate) characterization and microscopic observation. *Trichoderma* strains were sub-cultured from slants to PDA plates, and incubated at 28 °C for 48 h. After 2 days, when the colonies were visibly growing, but before conidial production, 5-mm diameter mycelia discs were cut from the actively-growing edge of the colony and were inoculated at the center of all of the freshly prepared PDA plates. Three replicates were maintained for each strain. 

Characteristics like colony appearance, growth rate, and sporulation patterns were recorded [31,32]. In order to assess the growth rate, the colony diameter of each strain growing on the PDA was measured at 24 h intervals, until the colony covered the plate.

### 2.3. Microscopic Study

Microscopic observation was carried out by following a water 3% KOH mount and using a slide culture technique stained with lactophenol cotton blue. After placing the cover slip, the slide was observed under the microscope for morphological characters, like conidiophores, their branching pattern, and angle to main axis; phialide numbers; their arrangement; conidial shape; color; formation of chlamydospores; and their position. Species identification was based on the morphological and taxonomic keys provided by Bisset [31,32]. All of the microscopic observations were made using an Olympus BX41 microscope (Olympus Life Sciences, Tokyo, Japan).

### 2.4. Molecular Identification 

#### 2.4.1. DNA Extraction, Amplification, and Sequencing

Sterilized cellulose membranes (Sigma) were plated on Petri dishes containing the PDA medium, and then inoculated with 100 μL of a suspension of 10^8^ spores per ml of each strain. The dishes were incubated at 28 °C for 24 h. The mycelium recovered from the membrane by scraping with a scratcher under sterile conditions was used to extract the DNA, according to the Hermosa method [24].

#### 2.4.2. Polymerase Chain Reaction (PCR) Purification and Sequencing

The ITS region of the nuclear rDNA gene cluster was amplified using Forward primer ITS1 (5′-TCCGTAGGTGAACCTGCGG-3′) and Reverse primer ITS4 (5′-TCCTCCGCTTATTGATATGC-3′) [34].

PCR-amplification reactions were performed in a 50 µL mixture, containing 5 μL of buffer (10×), MgCl_2_ (50 mM), 0.3 μL dNTPs (2 mM/μL), 2 μL of each primer (10 pM), 1 μL of Taq DNA polymerase (Biotools B&M Lab., Madrid, Spain) (5U), 1.5 μL (20 ng) of genomic DNA, and 38.2 μL of ultra-pure H_2_O. The reaction was conducted in a thermocycler (MJ Research, Bio-Rad Laboratories, Hercules, CA, USA) according to the following program: 3 min initial denaturation at 94 °C, followed by 35 cycles of 1 min denaturation at 94 °C, primers alignment at 53 °C for 1 min 30 s, primers extension to 72 °C for 2 min and final primers extension to 72 °C for 7 min. 

For the amplification of the *tef1* region, we used the same reaction mixture with Forward primer EF1–728f (5′-CATCGAGAAGTTCGAGAAGG-3′) and Reverse primer TEF1LLEr (5′-AACTTGCAGGCAATGTGG-3’) [22,35], according to the following program: 5 min initial denaturation at 94 °C, followed by 30 cycles of 1 min denaturation at 94 °C, primers annealing for 1 min at 58.1 °C, primers extension for 50 s at 74 °C, and a final primers extension for 7 min at 74 °C [1,28].

The PCR products were detected using 1% agarose gel electrophoresis and purified according to the gel extraction (NucleoSpin Extract II, Macherey-Nagel, Düren, Germany) clean-up kit protocol. The sequencing was carried out in both directions at the Department of Microbiology and Genetics of the University of Salamanca (Salamanca, Spain). The last 15 bases with a quality score value (Q) below 10 were pruned with the help of the publicly available Chromas 2.6.6 software. Molecular analysis was done by consensus sequences obtained using Bioedit v7.2.5 [36]. For molecular species identification, the ITS and *tef1* sequences were submitted to BLAST interface analysis in NCBI, GenBank database (http://blast.ncbi.nlm.gov/), and deposited in GenBank with the accession numbers provided in Table S1.

### 2.5. Phylogenetic Analysis

The data sets used for phylogenetic analyses included nine species of *Trichoderma,* which were isolated from different sampling sites of Algeria (Figure 1), and 17 reference sequences were assembled from GenBank (NCBI), namely: 3 *T. afroharzianum*, 5 *T. atrobrunneum*, 4 *T. harzianum*, 4 *T. longibrachiatum,* and 1 *Nectria eustromatica,* which was selected as the outgroup taxon. The materials used for the phylogenetic analyses and the GenBank accession numbers of the sequences generated in this study are shown in Table S1.

For ITS, *tef1*, and ITS + *tef1*, the evolutionary history was inferred by the maximum likelihood method based on the Tamura–Nei and Hasegawa–Kishino–Yano models [37,38].

The multiple sequence analysis and construction of phylogenetic trees were performed using MEGA X [39], and 1000 bootstrap replicates were taken in order to examine the reliability of the interior branches and the validity of the trees obtained [40].

## 3. Results

### 3.1. Morphological Characterization

The morphological analysis of nine strains was compatible with the *Trichoderma* genus description according to the common taxonomic phenotypical criteria [31,32].

The colony characters (color and texture) of all of the strains are given in Table 1. Strains ALG01, ALG02, ALG03, ALG04, and ALG06 had a fast growth, and their texture was floccose, effuse. and irregular, with an olive-green color and on the reverse a yellow green. The other strain, ALG05, also grew rapidly and produced green conidia that formed densely over the center and in undulating concentric rings towards the colony edge, and no pustules formed.

A similar colony development was observed for ALG07 and ALG08. Both strains developed from well-defined concentric white mycelium and produced green rings of conidia. ALG09 developed abundant, cottony mycelium with yellow pigmentation that was formed densely over the center and in undulating concentric rings toward the edge of the colony, and no pustules formed.

The growth rate assessment was not found to be useful in the characterization of *Trichoderma* strains. On the PDA culture, all of the strains covered the Petri dish (90 mm) in three days.

**Figure 2:**
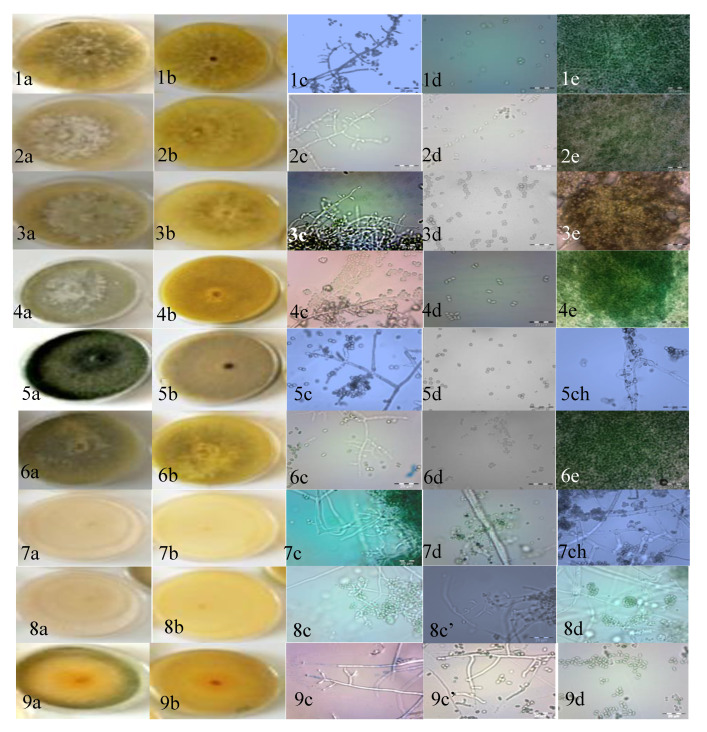
*Trichoderma* Potato Dextrose Agar (PDA) cultures. (**a**) Colony aspect after 5 days of growth at 28 °C, (**b**) reverse of colonies, (**c**) conidiophore, (**c’**) phialides, (**d**) conidia, (**e**) pustule and (**ch**) chlamydospores of different *Trichoderma* strains. (1–4, 6) *T. longibrachiatum*; (5, 9) *T. afroharzianum*; (7, 8) *T. atrobrunneum.* Scale bars in the microscopic photographs: 20 μm.

The results of the microscopic study revealed two groups of arrangements of conidiophores and phialides for the nine strains. The conidiophores of the first group (ALG01, ALG02, ALG03, ALG04, and ALG06) were characterized by the possession of long main branches, rarely re-branched, and the phialides. The phialides were either solitary or alternate and pale (Table 1 and Figure 2). With these characteristics, the *Trichoderma* strains were initially identified as *T. longibrachiatum*, according to the identification key described by earlier investigators [41,42,43].

The second group of strains (ALG05, ALG07, ALG08, and ALG09) was spread to the top and was smooth or rounded, and wide near the base. The phialides were arising mostly in a crowded manner, but had an angle with the conidiophore and had whorls of 2~5 on the terminal branches. The conidia were sub globose to ellipsoidal, and the apex was broadly rounded. The formation of chlamydospores was infrequent, and when they were formed they were terminal and intercalary (Table 1 and Figure 2). These descriptions are in agreement with the reports of earlier investigators [31,32,44]. Based on the results, strains ALG05, ALG07, ALG08, and ALG09 were identified as *T. harzianum*.

### 3.2. Molecular Analysis

The nine strains of this study were characterized by sequencing analysis and subsequent comparison with the databases containing ITS and *tef1* nucleotide sequences, with 584 bp and 600 bp in length, respectively.

The strains were identified via a BLAST search of GenBank, available in NCBI. For these searches, it is reasonable to start with ≥80% query coverage and ≥97−100% sequence similarity (i.e., up to 3% sequence divergence) to assign a name to the species based on consideration of the results from GenBank [45]. The NCBI GenBank accession numbers and isolation details are given in Table S1.

The results of BLAST analysis of the sequences of the ITS showed that five out of nine strains, ALG01, ALG02, ALG03, ALG04, and ALG06, were 100% identical to *T. longibrachiatum,* which corresponds to the results found in the microscopic study. The ALG07, ALG08, and ALG09 strains were 100% identical to *T. harzianum*, and ALG05 was 100% identical to *T. afroharzianum*. However, the ITS region is not considered appropriate on its own to delineate species in *Trichoderma,* as different species may share 100% ITS sequence identity [24,33,44].

The *tef1* sequence is preferred for this type of analysis because it contains introns, which allow for more variability between species. The *tef1* sequence provides a higher resolution than ITS at the species level [1,44]. The BLAST analysis of the *tef1* sequences established that the strains belonged to three different species (Table S1): *T. longibrachiatum*, *T. afroharzianum*, and *T. atrobrunneum*.

### 3.3. Phylogenetic Analysis

The phylogenetic analyses were conducted on the sequence data of ITS (584 bp), *tef1* (600 bp), and ITS + *tef1* (1184 bp). All of the nucleotide data were weighted equally, and gaps were treated as missing characters. After removing the ambiguously aligned regions, the alignments were 1184 characters, of which 213 were phylogenetically informative. The parsimony informative characters were 131 in the *tef1* alignment and 82 in ITS.

**Figure 3:**
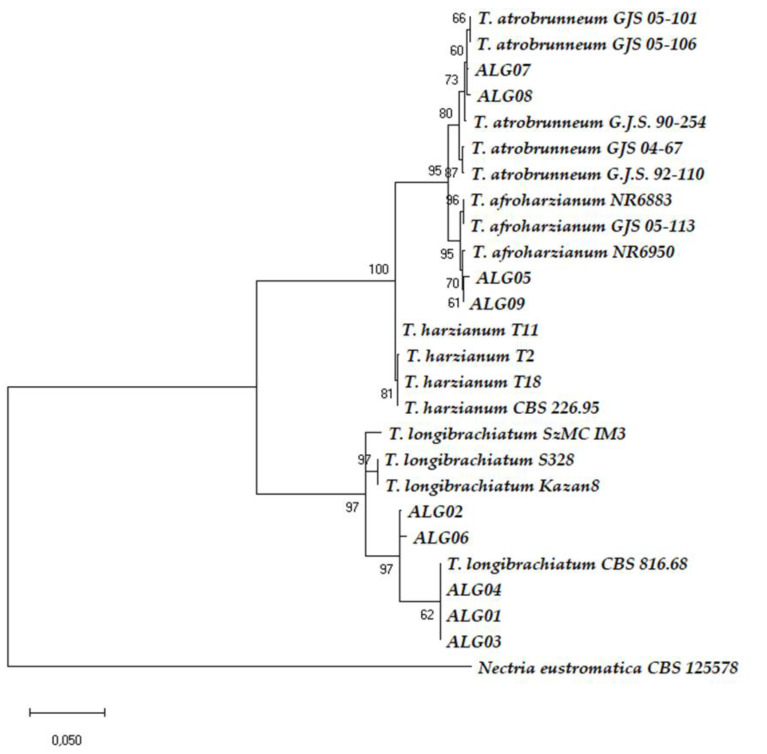
Phylogenetic tree of concatenated ITS + *tef1* sequences from 26 *Trichoderma* strains. The ITS + *tef1* sequences from *Nectria eustrimatica* was used as the out-group. The scale bar represents the number of expected substitutions per site.

A phylogram was constructed in order to understand the relationship between the strains and various species of *Trichoderma.* The phylogenetic trees (Figures S1, S2 and Figure 3) showed that all isolates were separated into two clades. The ALG01, ALG02, ALG03, ALG04, and ALG06 strains belonged to the Clade Longibrachiatum, whereas the ALG05, ALG07, ALG08, and ALG09 strains were located in the Clade Harzianum.

The phylogenetic analysis based on the ITS sequences of nine *Trichoderma* strains showed the five strains of the Clade Longibrachiatum were grouped with the *T. longibrachiatum* reference sequences: CBS 816.68, SzMC IM3, S328, and Kazan 8. On the other hand, the four strains belonging to the Clade Harzianum had a bootstrap support of 99%. However, these strains and the references were presented with an anarchic configuration, and the ALG09 strain could not be distinguished from reference sequences. ALG05 and ALG07 were clustered with *T. afroharzianum* GJS05–113 with a low bootstrap. We noticed that ALG08 was the most closely related between GJS 92–110 of *T. atrobrunneum* and NR6883 of *T. afroharzianum* (Figure S1).

To eliminate ambiguity and for the exactness of the species identification, we analyzed the *tef1* data (Figure S2). The *tef1* phylogenetic analyses showed that the Clade Harzianum had a high bootstrap support. The relationship between all of the reference isolates could be clearly distinguished at the level of species. In this case, there was not any ambiguity to distinguish species. So, the ALG07 and ALG08 strains were identified as *T. atrobrunneum* (bootstrap support 86%), and ALG05 and ALG09 were identified as *T. afroharzianum* (bootstrap support 95%; Figure S2).

The combination of multi-loci sequences based on a phylogenic tree analysis has been suggested for the better identification of *Trichoderma* spp. [46,47]. Therefore, a phylogenetic tree was built using sequences of the two loci of ITS + *tef1* to get 5’ITS-*tef1*–3’ concatenated sequences. We found almost the same results as in the *tef1* phylogenic analyses, but the bootstraps were resolved with a better bootstrap support (Figure 3).

## 4. Discussion 

In the present work, a total of nine strains belonging to two clades of *Trichoderma* were isolated from different sampling sites of Algeria. Species identification was based on two DNA gene sequencing analysis, including ITS- and *tef1*-based NCBI BLAST analysis (Table S1 and Figure 3). We chose this molecular approach because the study of the morphological and cultural characteristics (Table 1) could not efficiently distinguish *Trichoderma* strains up to the species level. Phenotypic traits are not constant and are influenced by culturing conditions [20], secondary metabolite production [6], and, particularly in *Trichoderma*, they can often be misleading because of the incorporation of many types of genes by horizontal gene transfer and mycoparasitism from plant-associated filamentous fungi belonging to different phylogenetically close classes of Ascomycota hosts [48].

The combined analysis of ITS and *tef1* sequences is more advantageous for the identification of species [1], as a single sequence analysis like ITS is also not enough to molecularly identify closely related species [49,50]. The ITS BLAST results showed that strains ALG05, ALG07, ALG08, and ALG09 were located in *T. harzianum*, but this was not enough for species delimitation, as ca. 27% of all fungal ITS sequences from GenBank were submitted with insufficient taxonomic identification, and 20% of the fungal sequences in this data base may be incorrectly annotated [51,52]. It has been pointed out that an analysis based on ITS sequences is not able to distinguish closely related species within *Trichoderma* species complexes such as Harzianum or Longibrachiatum [53].

Our nine strains of *Trichoderma* were involved in the ITS phylogenetic study, and the resulting tree constructed by maximum likelihood analysis showed an overall poor resolution for most of the strains analyzed (Figure S1). These results revealed the presence of low intraspecific variations between the investigated ITS *Trichoderma* sequences. Similar findings on other fungi proved that ITS was not the appropriate gene marker to discriminate between the members of the genera *Aspergillus, Cladosporium*, *Fusarium*, *Penicillium,* or *Trichoderma*, as these taxa have narrow or no barcode discrepancies in their ITS regions [45,54].

Our study supports that *tef1* sequencing is significantly more useful than the ITS for species delimitation in *Trichoderma*, particularly when attempting to identify those within the *T. harzianum* complex. Fanelli et al. (2018) suggested that the combination of multi-loci sequences based on the analysis of phylogenic trees gives a better distribution of *Trichoderma* spp. [46]. The analysis of concatenated ITS + *tef1* sequences allowed these authors to reclassify the *T. harzianum* ITEM 908 as *T. atrobrunneum*, a new species that was split up with *T. afroharzianum* and *T. guizhouense* from the *T. harzianum* complex [46]. Other previous works reported similar observations [47,55]. In our approach, phylogenetic analyses of *tef1* and of the ITS + *tef1* concatenation served to provide a more conclusive identification at the Clade Harzianum level, where the members of this clade were phenotypically and genotypically very similar. These phylogenetic analyses made it possible to correct and attribute the affiliation of ALG07 and ALG08 as *T. atrobrunneum*, and ALG05 and ALG09 as *T. afroharzianum.* These two species have been split off from the *T. harzianum* complex [44]. In fact, *T. afroharzianum* and *T. atrobrunneum* were considered (and some strains were registered for commercial use) as *T. harzianum*. Interestingly, *T. harzianum* T22, probably the best-known *Trichoderma* commercial strain [3], has been reallocated within *T. afroharzianum* [44]. The bootstrapping values were low at the level of the ITS tree, and this gene marker, the most universal fungal molecular barcode, was not appropriate to discriminate between the members of the Harzianum clade. It should be noted that the name T. harzianum refers to an old single species that grouped strains of the today’s known *T. afroharzianum* and *T. atrobrunneum,* which are very closely related new species. It can be confusing that an important part of the members of the old *T. harzianum* species complex are still called T. harzianum. A correct identification is particularly important for those who want to register active matters with commercial purposes.

*T. atrobrunneum* is found in soil or on decaying wood, clearly or cryptically parasitizing other fungi, and *T. afroharzianum* is known mostly from soil, but can be isolated from roots and other fungi [44]. *T. atrobrunneum* has been previously found in North America, Spain, France, Italy Croatia, and Greece [27,44], and the present study describes its presence in Africa. *T. afroharzianum* is a widespread fungus that can grow well at 35 ºC, so it is not strange to find it in Algeria. 

In our study, the phylogeny carried out by ITS + *tef1* confirmed the same results obtained by *tef1* (Figure 3 and Figure S2). and served to allocate strains ALG01, ALG02, ALG03, ALG04, and ALG06 to *T. longibrachiatum*. AGL02 and AGL06 were located within the core group of *T. longibrachiatum* strains in Bayesian phylograms built for species delimitation within the Clade Longibrachiatum [56]. Strains AGL01, AGL03, and AGL04 showed identical *tef1* sequence (EU401591) than that of the reference strain *T. longibrachiatum* CBS 816.68 (ATCC 18648), separated from the central core in the Bayesian phylograms, but belonging to *T. longibrachiatum* [42,56]. *T. longibrachiatum* is a cosmopolitan and predominantly tropical species that has been previously isolated in Egypt and Ghana [56,57], although it is described in Algeria for the first time in this report.

Of the three species identified in our analysis, *T. longibrachiatum* was isolated from three sites, while *T. afroharzianum* and *T. atrobrunneum* were isolated from a single different site. In any case, green soil debris with mycelial masses were observed for the three *Trichoderma* spp., this being indicative of the active growth and sporulation of the *Trichoderma* strains in the natural environments where they were sampled. According to Chaverri et al. [44], phylogenetic analyses seem to be linked to a certain segregation according to geographic sampling. Although no global study of the distribution of *Trichoderma* species can follow the rapid taxonomic change, recent reassessments of existing international culture collections and new collection efforts in sub-sampled geographic areas have significantly expanded our knowledge [50].

*Trichoderma* belongs to the kind of fungal genera that constantly reshape their genome for rapid responses and successful competition in potentially new habitats, thus becoming environmental opportunists [58]. Moreover, the ability to endoparasitise closely related species (up to adelphoparasitism) could favor host/parasite DNA exchanges, and further contribute to the formation of the unique core genome of *Trichoderma* [48,58]. It has been argued that the versatility of *Trichoderma* nutritional strategies can be described by the expansions of the spectrum of hosts and substrates due to the enrichment of its genome by the laterally transferred genes required for feeding on the plant biomass [48,58]. Du Plessis et al. [59] explains the unusual occurrence of fungal species in Africa and southern regions due to insufficient research efforts in order to shed light on the vast and wide range of fungal populations in more than half of the world’s ecosystems. Obviously, further efforts are needed, and the comprehensive understanding of this important genus will be to focus in more detail on the specific ecological niches and physicochemical needs of different species, using undoubtedly metabarcoding approaches.

## 5. Conclusions

Despite the small number of samples (nine strains), an integrated taxonomical approach enabled us to report, for the first time, three *Trichoderma* species in Algeria, namely, *T. atrobrunneum* (south), *T. longibrachiatum* (south), and *T. afroharzianum* (northwest), describing the presence of *T. atrobrunneum* in Africa. This confirms that the diversity of geographic environments and the types of climate in Algeria offer the possibility of survival for different types of *Trichoderma*.

Unfortunately, the diversity of the *Trichoderma* species remains relatively less studied in Africa, and in Algeria in particular, compared with other parts of the world. It is important for future studies to expand the sampling areas and increase the number of studies that will better reflect the overall situation of *Trichoderma* biodiversity in Algerian, and by extension in African ecosystems, by metabarcoding high-throughput sequencing efforts.

## Figures and Tables

**Table 1:** Morphological description used for characterization of nine *Trichoderma* strains.

Groups	Strains	Colony			Conidia				Phialides		
		Color	Reverse color	Edge	Conidiation	Shape	Size (µm)	Color	Shape	Size (µm)	Disposition
Group 1	ALG01	Light green	Yellowish green	Wavy	Concentric zones	Ellipsoidal, subglobose	4.2–6.2 × 2.45–3.0	Pale green	Globose	7–10.5 × 2.5–3.0	Solitary
	ALG02	Dark green	Light yellow	Wavy	Ring like Zones	Ellipsoidal, subglobose	4–6 × 3.0–4.0	Green	Bottle shape	6.9–11.1 × 2.5–3.0	Solitary
	ALG03	Dark green	Light yellow	Wavy	Concentric zones	Ellipsoidal, subglobose	4–6.2 × 2.4–3.2	Green	Bottle shape	6–11.5 × 2.5–2.9	Solitary
	ALG04	Light green	Light yellow	Wavy	Ring like Zones	Ellipsoidal, Globose	4–6 × 3–4	Green	Bottle shape	6–11.2 × 4–2.9	Solitary
	ALG06	Light green	Light yellow	Wavy	Concentric zones	Ellipsoidal, Globose	4–6 × 2.4–3.2	Light green	Bottle shape	6–11.4 × 2.5–3	Solitary
Group 2	ALG07	White to green	Light yellow	Smooth	Broad concentric rings	Subglobose to ovoid	3.2–3.5 × 2.8–3.0	Dark green	Ampulliform to lageniform	4.2–7.3 × 2.8–3.7	Tending clustered 2–5 whorls
	ALG08	White to green	Light yellow	Smooth	Broad concentric rings	Subglobose to ovoid	3.2–3.5 × 2.8–3.0	Light green	Ampulliform to lageniform	3.9–7.1 × 2.5–3.4	Tending clustered, 2–5 whorls
	ALG05	Dark green	Creamy	Smooth	Concentric rings	Subglobose to ovoid	2.5–3.2 × 2.2–2.8	Light green	Lageniform to ampulliform	4.7–7.9 × 2.3–3.5	Tending clustered, 2–5 whorls
	ALG09	Yellowish green	Light yellow	Smooth	concentric rings	Subglobose to ovoid	2.7–3.5 × 2.5–3.2	Light green	Lageniform to ampulliform	4.8–8.0 × 2.4–3.6	Tending clustered, 2–5 whorls

## Supplementary Materials

The following are available online at https://www.mdpi.com/2076-2607/8/10/1455/s1, Figure S1. Phylogenetic tree of internal transcribed spacer (ITS) sequences from 26 *Trichoderma* strains. The ITS sequence from *Nectria eustrimatica* was used as the out-group. The scale bar represents the number of expected substitutions per site, Figure S2. Phylogenetic tree of translation elongation factor (*tef1*) sequences from 26 *Trichoderma* strains. The *tef1* sequence from *Nectria eustromatica* was used as the out-group. The scale bar represents the number of expected substitutions per site, Table S1. *Trichoderma* strains used in the phylogenetic analyses as well as their corresponding GenBank accession numbers.
