# Supplementary material for: Three New Reports of Trichoderma in Algeria: T. atrobrunneum, (South) T. longibrachiatum (South), and T. afroharzianum (Northwest)
Source: Microorganisms. 2020 Sep 23;8(10):1455. doi: 10.3390/microorganisms8101455 (PMC7597948; doi:10.3390/microorganisms8101455)
Supplement: Supplementary file 1 [file microorganisms-08-01455-s001.pdf]

Supplementary Materials

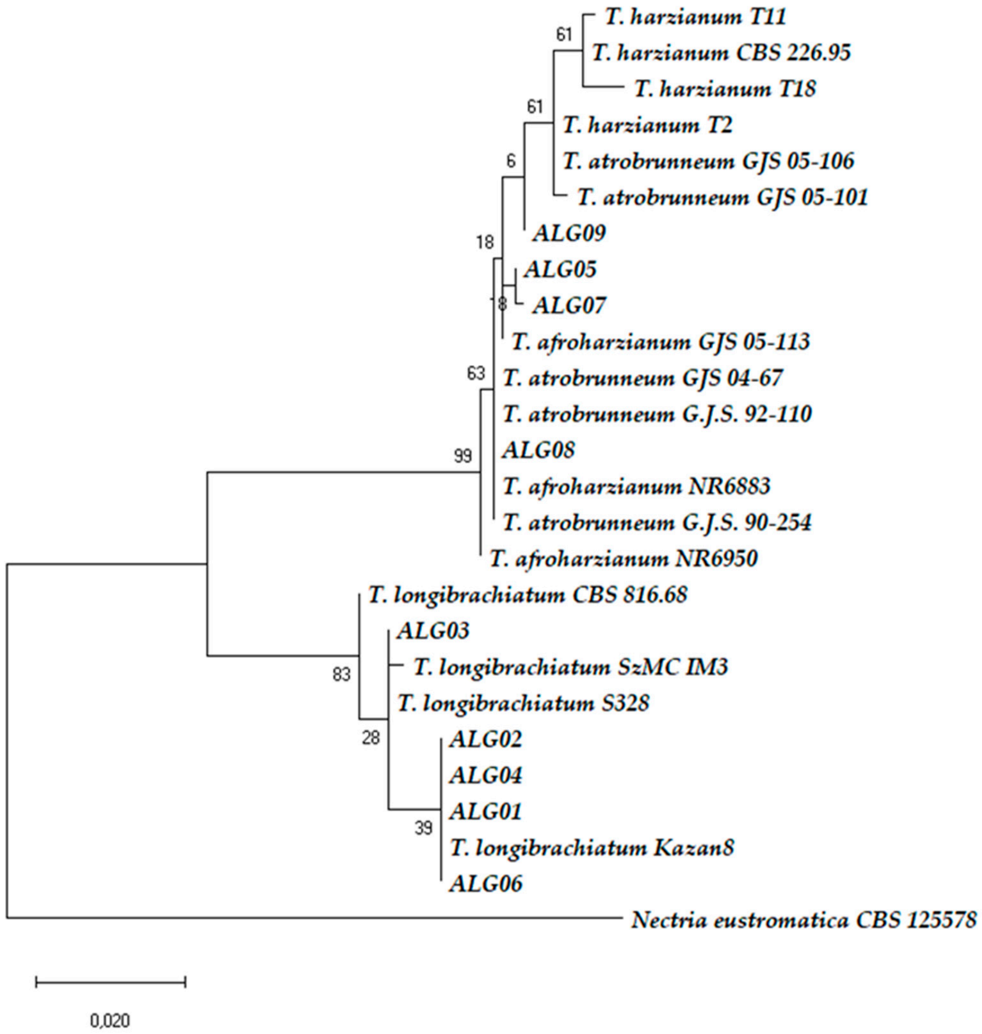

Figure S1. Phylogenetic tree of internal transcribed spacer (ITS) sequences from 26 *Trichoderma* strains. The ITS sequence from *Nectria eustrimatica* was used as the out-group. The scale bar represents the number of expected substitutions per site.

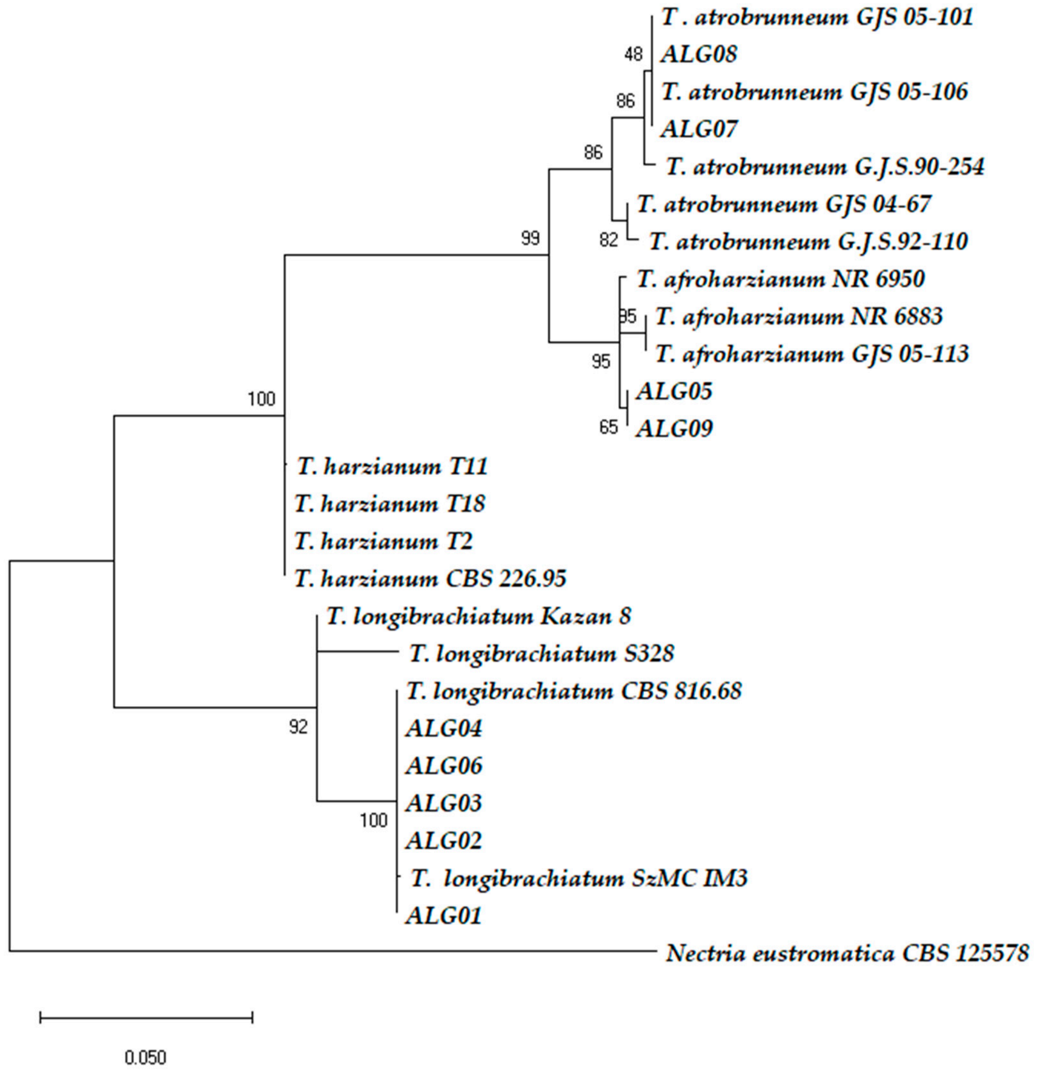

Figure S2. Phylogenetic tree of translation elongation factor (*tef1*) sequences from 26 *Trichoderma* strains. The *tef1* sequence from *Nectria eustromatica* was used as the out-group. The scale bar represents the number of expected substitutions per site.

Table S1. *Trichoderma* strains used in the phylogenetic analyses as well as their corresponding GenBank accession numbers.

Species	Strain ID	Origin	Habitat	GenBank accession number	
				ITS	*tef1*
T. afroharzianum	NR6883	Japan	Soil	AF194010	AF348091
T. afroharzianum	NR 6950	Japan	Soil	AF194015	AF348105
T. afroharzianum	**ALG05**	**Algeria**	**Soil**	**MT000970**	**MT472845***
T. afroharzianum	**ALG09**	**Algeria**	**Soil**	**MT000974**	**MT472849***
T. afroharzianum	G.J.S. 05-113	Italy	Wheat Seed	FJ442235	FJ463378
T. atrobrunneum	G.J.S. 04-67	Italy	Soil	FJ442273	FJ463360
T. atrobrunneum	G.J.S.92-110	France	Wood	AF443924	AF443942
T. atrobrunneum	G.J.S.90-254	Germany	NA	AF443926	AF443943
T. atrobrunneum	GJS 05-101	Italy	Soil	FJ442677	FJ463392
T. atrobrunneum	G.J.S.05.106	Italy	Soil	FJ442678	FJ463297
T. atrobrunneum	**ALG07**	**Algeria**	**Soil**	**MT000972**	**MT472847***
T. atrobrunneum	**ALG08**	**Algeria**	**Soil**	**MT000973**	**MT472848***
T. harzianum	T18	NA	NA	KX632492	KX632606
T. harzianum	T11	NA	NA	KX632486	KX632600
T. harzianum	T2	Peru	*Hevea brasiliensis*	FJ884174	KX632591
T. harzianum	CBS 226.95	UK	Soil	AY605713	AF348101
T. longibrachiatum	S328	Spain	Wood	JQ685875	JQ685867
T. longibrachiatum	CBS 816.68	USA	Mud in creek	EU401556	EU401591
T. longibrachiatum	Kazan 8	Russia	Soil	EU401561	EU401610
T. longibrachiatum	SzMC IM3	NA	Human nose	EU401576	EU401627
T. longibrachiatum	**ALG01**	**Algeria**	**Soil**	**MT000966**	**MT472841***
T. longibrachiatum	**ALG02**	**Algeria**	**Soil**	**MT000967**	**MT472842***

T. longibrachiatum	**ALG03**	**Algeria**	**Soil**	**MT000968**	**MT472843***
T. longibrachiatum	**ALG04**	**Algeria**	**Soil**	**MT000969**	**MT472844***
T. longibrachiatum	**ALG06**	**Algeria**	**Soil**	**MT000971**	**MT472846***
Nectria eustromatica	CBS 125578	Italy	*Hippocrepis emerus*	HM534897	HM534876

NA: not available, *Trichoderma* strains isolated from this study are indicated in boldface. *Numbers in bold indicate newly submitted sequences.
